# Detection of Acquired Antibiotic Resistance Genes in Domestic Pig (*Sus scrofa*) and Common Carp (*Cyprinus carpio*) Intestinal Samples by Metagenomics Analyses in Hungary

**DOI:** 10.3390/antibiotics11101441

**Published:** 2022-10-20

**Authors:** Balázs Libisch, Sahabi Abdulkadir, Tibor Keresztény, Péter P. Papp, Ferenc Olasz, Hedvig Fébel, Zsuzsanna J. Sándor, Geertrui Rasschaert, Ellen Lambrecht, Marc Heyndrickx, András Szabó, Melinda Kovács, Katalin Posta

**Affiliations:** 1 Agribiotechnology and Precision Breeding for Food Security National Laboratory, Institute of Genetics and Biotechnology, Hungarian University of Agriculture and Life Sciences, 2100 Gödöllő, Hungary; 2 Bawu Veterinary Consult, Jalingo 660213, Nigeria; 3 Doctoral School of Biological Sciences, Hungarian University of Agriculture and Life Sciences, 2100 Gödöllő, Hungary; 4 Agribiotechnology and Precision Breeding for Food Security National Laboratory, Nutrition Physiology Research Group, Institute of Physiology and Nutrition, Hungarian University of Agriculture and Life Sciences, 2053 Herceghalom, Hungary; 5 Research Centre for Aquaculture and Fisheries (HAKI), Institute of Aquaculture and Environmental Safety, Hungarian University of Agriculture and Life Sciences, 5541 Szarvas, Hungary; 6 Technology & Food Science Unit, Flanders Research Institute for Agriculture, Fisheries and Food, 9090 Melle, Belgium; 7 Agribiotechnology and Precision Breeding for Food Security National Laboratory, Institute of Physiology and Nutrition, Hungarian University of Agriculture and Life Sciences, 7400 Kaposvár, Hungary; 8 ELKH-MATE Mycotoxins in the Food Chain Research Group, Hungarian University of Agriculture and Life Sciences, 7400 Kaposvár, Hungary

**Keywords:** intestinal microbiome, resistome, antibiotic resistance, One Health, metagenomics, food safety, carbapenemase

## Abstract

The aim of this study was metagenomics analyses of acquired antibiotic-resistance genes (ARGs) in the intestinal microbiome of two important food-animal species in Hungary from a One Health perspective. Intestinal content samples were collected from 12 domestic pigs (*Sus scrofa*) and from a common carp (*Cyprinus carpio*). Shotgun metagenomic sequencing of DNA purified from the intestinal samples was performed on the Illumina platform. The ResFinder database was applied for detecting acquired ARGs in the assembled metagenomic contigs. Altogether, 59 acquired ARG types were identified, 51 genes from domestic pig and 12 genes from the carp intestinal microbiome. ARG types belonged to the antibiotic classes aminoglycosides (27.1%), tetracyclines (25.4%), β-lactams (16.9%), and others. Of the identified ARGs, *tet*(E), a *bla_OXA_*-_48-like_ β-lactamase gene, as well as *cphA4*, *ampS*, *aadA2*, *qnrS2*, and *sul1*, were identified only in carp but not in swine samples. Several of the detected acquired ARGs have not yet been described from food animals in Hungary. The *tet*(Q), *tet*(W), *tet*(O), and *mef*(A) genes detected in the intestinal microbiome of domestic pigs had also been identified from free-living wild boars in Hungary, suggesting a possible relationship between the occurrence of acquired ARGs in domestic and wild animal populations.

## 1. Introduction

The discovery of antibiotics resulted in a significant reduction in the mortality and morbidity rates of infectious diseases, both in humans and in animals. However, with the worldwide spread of intensive farming practices involving significant antimicrobial use, associations were reported between the consumption of an antimicrobial class and the corresponding bacterial resistance both in food-producing animals and in humans [1,2,3,4].

The economic importance of domestic pig production in Hungary is indicated by the proportion of pork within the overall national meat consumption, which was stably between 40 and 45% in the 2000–2015 period based on KSH (Hungarian Central Statistical Office) data [5]. Regarding common carp, the leading carp-producing country in the European Union in 2015 was the Czech Republic with 20,900 metric tons of production, closely followed by Hungary with 18,000 metric tons [6].

The European Green Deal Farm to Fork Strategy established a target to reduce the sales of antimicrobials by 50% for farmed animals and in aquaculture by 2030. Among the 25 countries that have reported to the European Surveillance of Veterinary Antimicrobial Consumption (ESVAC) every year since 2011, the overall sales of antimicrobials decreased by 43.2% during 2011–2020. As a result, when assessed per kg biomass, the antimicrobial consumption in the EU was overall lower in food-producing animals than in humans between 2016 and 2018 [4,7,8].

The spread of antibiotic resistance genes (ARGs) requires the transfer and acquisition of genetic elements encoding antibiotic resistance determinants between bacterial strains or species. The resistant bacterial populations can transmit their ARGs to their progeny through vertical evolution or to other bacterial strains and species through horizontal transfer [9]. The genetic density and complexity of the gut microbial community stimulate the spread of ARGs among microbes [10]. The intestinal tracts of food animals are important ARG reservoirs. However, ARGs can also be present in healthy animals, regardless of their prior antimicrobial exposure [11]. Manure from animals that have not received antibiotics may also contain high numbers of ARGs [12], and ARGs can persist on farms for many years after the abolishment of antibiotic use [13]. These observations suggest that other factors such as historical antimicrobial use and farm management practices should also be considered to explain variations in antimicrobial resistance (AMR) [14].

Aquatic environments may also become reservoirs of antimicrobial-resistant bacteria from various sources, such as wastewaters, hospital effluents, and animal or plant agricultural run-off. Antibiotics may be administered to treat or prevent the spread of infectious diseases in aquaculture as well [15]. Antibiotics in aquaculture are mainly administered through feed or immersion or by direct application into water [16,17]. Some studies indicated that approximately 70–80% of the antimicrobials applied in aquaculture are dispersed into water systems [18].

In June 2017, the European Union announced a new action plan to combat the spread of AMR: the European One Health Action Plan against Antimicrobial Resistance [19]. The objectives of this action plan include research to understand the epidemiology of AMR, closing knowledge gaps on AMR in the environment and on how to prevent transmission of AMR between animals and humans.

Land application of animal manure that contains resistant bacteria and antibiotics may facilitate the establishment of an environmental reservoir of antibiotic-resistant bacteria [12,20,21], thereby promoting further potential dissemination into wild animal populations as well [22]. ARG abundance in feces of people working in pig/broiler farms was also related to livestock AMR, possibly by exposure to animal feces and dust in farms, suggesting that antimicrobial residues in farm dust may also impact the gut microbiota by selecting for resistance [23].

Current surveillance approaches are primarily focusing on a limited number of zoonotic pathogens from animal feces or food, such as *Salmonella* spp., *Campylobacter jejuni*, and *Escherichia coli* [24]. These surveillance approaches may be further facilitated by applying next-generation sequencing (NGS) techniques that have revolutionized the analysis of complex bacterial communities as well through metagenomic analyses [25,26]. Metagenomic methods can overcome the limitations of culture-based approaches as the entire repertoire of resistance genes (the resistome) can be assessed in a sample without culturing single isolates of pre-selected species, which may cause a loss of relevant information [7]. Metagenomics may also contribute to the identification and characterization of non-culturable, difficult-to-culture, or slow growing microorganisms and be used for the tracking of genetic determinants of AMR or virulence [27,28]. Since shotgun metagenomic sequencing facilitates the detection of a wider range of ARGs in complex bacterial communities compared to conventional PCR-based approaches, it may contribute to the tracing of ARGs between different habitats and environments and to identifying potential routes of dissemination [7,25,26]. Although the de novo assembly of NGS reads into metagenomic contigs may result in the loss of some low abundance data, their assembly into contigs allows for a more accurate detection and identification of ARGs and potentially the exploration of their immediate genetic environment [26,29].

Munk et al. [30] quantified and characterized the acquired fecal resistomes of 181 pig and 178 poultry farms from nine European countries using shotgun metagenomics. In pig fecal samples the tetracycline, macrolide, β-lactam, and aminoglycoside ARGs were the most common AMR types, whereas other types of AMR were comparatively rare [30]. Among the main AMR determinant classes, positive associations were found between antimicrobial use vs. ARG relative abundance for macrolides and tetracyclines in a cross-sectional study among 176 conventional pig farms from nine European countries. On the other hand, no similar associations were demonstrated between colistin and aminopenicillin use and the relative abundance of the corresponding ARG classes [31].

To the best of our knowledge, the occurrence of acquired ARGs in domestic pig and common carp intestinal samples has not yet been reported from Hungary using shotgun metagenomic methods. The aim of this pilot study was metagenomics analyses of acquired antibiotic resistance genes in the intestinal microbiome of these two important food animal species in Hungary from a One Health perspective and comparative analysis with results of national and international studies of a similar scope. By use of an up-to-date metagenomic approach, a broad variety of acquired antimicrobial resistance determinants and associated mobile genetic elements (MGEs) were demonstrated in the microbiome of domestic pig and common carp intestinal samples from Hungary, including ARGs for two critically important antimicrobial classes, the quinolones and carbapenems. Monitoring and understanding the distribution and ways of dissemination for acquired antimicrobial resistance determinants is necessary for combating AMR both by national and European mitigation strategies.

## 2. Results

### 2.1. Taxonomic Assignment of the Metagenomic Contigs to Bacterial Families

Since the presence of acquired ARGs was explored in this study at the contig level, taxonomic annotations of the obtained swine- and carp-gut metagenomic contigs were performed by Kraken against its standard “Bacteria” database.

**Figure 1:**
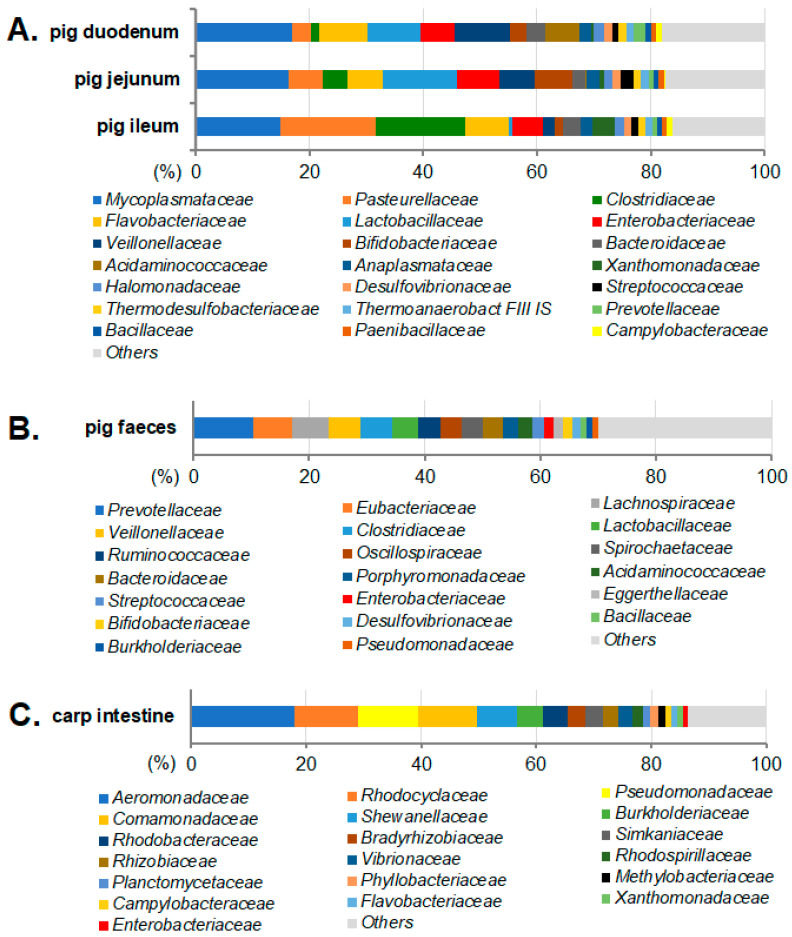
Mean proportions (%) of different bacterial families among metagenomic contigs classified to the kingdom of Bacteria. Bacterial families assigned to metagenomic contigs for the swine small intestine (**A**), for swine feces (**B**) and for the carp intestine (**C**).

Results for taxonomic annotations of the assembled metagenomic contigs are summarized in Figure 1. Bacterial families containing potentially pathogenic and/or opportunistic pathogenic bacteria (such as the *Enterobacteriaceae*, *Pseudomonadaceae*, *Aeromonadaceae*, *Streptococcaceae*, *Campylobacteraceae*, and *Enterococcaceae*) were represented in the assembled metagenomic contig sets with variable and substantial proportions. On the other hand, families generally containing commensal gut bacteria, including the *Ruminococcaceae*, *Lachnospiraceae*, *Lactobacillaceae*, *Bacillaceae*, *Bifidobacteriaceae*, *Rikenellaceae*, and other families, were also represented by the contigs, making it possible to assess the ARG repertoire not only of the commonly examined pathogenic species but also of the commensal bacterial community of the intestinal microbiota. The metagenomic contigs of the pig small intestine samples (duodenum, jejunum, and ileum) were annotated in higher mean proportions to certain bacterial families compared those of the pig feces samples, in particular for the *Mycoplasmataceae*, *Pasteurellaceae*, and *Flavobacteriaceae* (Figure 1).

### 2.2. Identification of Acquired ARG Types in Domestic Pig Intestinal Microbiome

**Figure 2:**
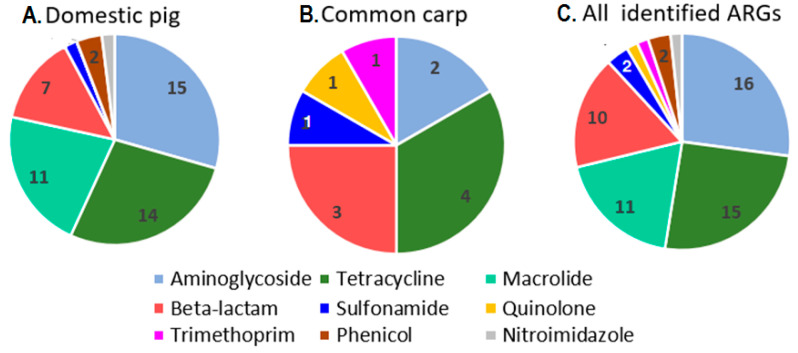
The number of ARG types assigned to different antibiotic classes. (**A**) Domestic pig intestinal samples, (**B**) common carp intestine, and (**C**) all samples analyzed. Pie chart sections without a number contain one ARG type. ResFinder antibiotic classes were applied according to Munk et al. (2018) and Van Gompel et al. (2019) [30,31].

The various acquired ARG types detected in the domestic pig and common carp intestinal samples using ABRricate and the ResFinder 4.1 database are summarized in Table 1, and additional associated data are provided in Supplementary Tables S1–S4. In total, 51 different ARG types were detected in the swine gut microbiome, out of which 15 genes (29.4%) conferred resistance against aminoglycosides, 14 genes (27.4%) against tetracyclines, 7 genes (13.7%) against β-lactams, and 15 genes against other antibiotic classes (Figure 2A).

Of the 51 identified ARG types, 44 genes (86.2%) were detected in feces, 38 genes (74.5%) in the small intestine, 12 genes (23.5%) solely in feces, and 6 genes (11.7%) solely in the small intestine (Table 2). Their coverages ranged from 60% to 100% in accordance with the applied ABRicate parameter settings, where 100% query coverage was obtained for 42 ARG types from swine. The percentage identity with the ResFinder reference sequences was ≥95% for all ARGs reported in this work, making the analysis more stringent than the >90% identity threshold recommended by Lal Gupta et al. (2020) and Doyle et al. (2020) [26,32]. Identity cutoffs > 90% were suggested to increase the probability of targeting genes that are actually functional ARGs [26].

The following ARG types were demonstrated in all four examined swine intestinal segments (duodenum, jejunum, ileum, and feces): *tet*(Q), *ant(6)-Ia* (that is, *aadE*), *aph(2″)-If*, *bla*_ACI-1_, *mef*(A), and *lnu*(C). Moreover, *tet*(W) was harbored by contigs from all three segments of the small intestine with 94.84% to 100% coverage (Supplementary Table S1), whereas it was also detected on a swine fecal contig with 55% coverage, which is only slightly lower than the 60% coverage threshold applied in this study.

### 2.3. Immediate Genetic Environment of ARGs on Selected Swine Gut Metagenomic Contigs

**Figure 3:**
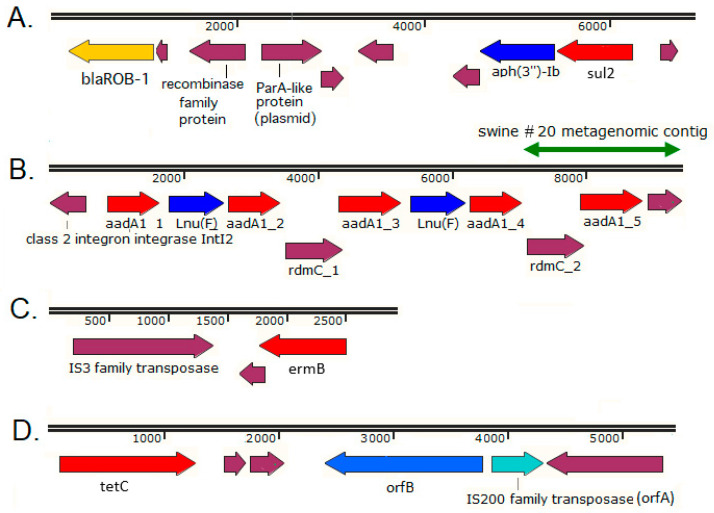
The immediate genetic environment of the acquired ARGs on selected swine gut metagenomics contigs. Metagenomic contigs from the ileum of swine #12 (**A**), the feces of swine #20 (**B**), the jejunum of swine #2 (**C**), and the jejunum of swine #18 (**D**). The green arrow indicates the 1704 bp region of the *E. coli* plasmid p702_18_1 that is covered by the swine #20 metagenomic contig (**B**).

As summarized in Supplementary Table S6, the mean length of metagenomic contigs (in the range of 512 bp–960 bp) was not sufficient to provide information about the genetic context of most identified ARGs; however, in certain cases their genetic context was available. A gene encoding a ROB-1 β-lactamase was detected in the ileum of swine #12 (Supplementary Tables S3 and S5), harbored by a contig that also carried an *aph(3″)-Ib* (that is, *strA*) aminoglycoside phosphotransferase gene and a *sul2* dihydropteroate synthase gene (Figure 3A). This contig showed 99.9% identity in a 6392 bp region (containing these three ARGs) with the *Actinobacillus porcitonsillarum* (*Pasteurellaceae*) pKMA202 plasmid (NCBI Accession: AM748706 [33]).

In *A. porcitonsillarum*, the *bla*_ROB-1_ gene was also present on the same genetic element with the *sul2* and *strA* genes, whereas *bla*_ROB-1_ usually appeared alone on *A. pleuropneumoniae* plasmids and on other *Pasteurellaceae* plasmids, such as ROB-1 plasmids of *Pasteurella haemolytica*, plasmids pAB2 of *Mannheimia haemolytica*, RRob from *Haemophilus influenzae*, and pB1000 from *Haemophilus parasuis* [33]. Two additional genes (*parA*, *res*) that may be involved in plasmid maintenance and propagation were also localized in all larger *A. porcitonsillarum* plasmids. The *parA* gene is also located on the complementary strand of pKMA202. The encoded 229-amino-acid sequence revealed homology to ParA-like partitioning proteins essential for plasmid stability [34].

An *aadA1* (streptomycin 3″-adenylyltransferase) gene was carried by a 1733 bp contig from the feces of swine #20 (Supplementary Tables S2 and S5, Figure 3B) that proved to be 99.9% (1703/1704 bp) identical with the corresponding 1704 bp region of the *E. coli* strain 702/18 plasmid p702_18_1, which is a 219 kbp IncHI2A plasmid isolated from a human rectal swab in 2018 in Germany (NCBI Accession: NZ_CP074702, [35]). This 1704 bp region of the plasmid contains the fifth copy from an array of five *aadA1* genes, where four of the five *aadA1* genes were identical, but the fifth copy contained a silent SNP at position 672 and a codon deletion at position 689–691 (Glu230) (Figure 3B, [35]). The metagenomic contig obtained from the feces of swine #20 contained an *aadA1* gene identical to this fifth variant copy of *aadA1* on plasmid p702_18_1, together with its immediate genetic context (Figure 3B).

An erythromycin resistance determinant *erm*(B) gene, encoding a ribosomal RNA methyltransferase, was harbored by a metagenomic contig of 2926 bp from the jejunum of swine #2 (Supplementary Tables S4 and S5, Figure 3C) that showed 99.2% identity at 92% coverage with the corresponding segment of the *Lactobacillus crispatus* CHCC3692 strain transposon Tn*3692* (NCBI Accession: AY262353, [36]). The *L. crispatus* CHCC3692 strain was originally cultured from the esophagus of suckling pigs in Karlebo, Denmark, in 1987. The examined transposon region contained the *erm*(B) gene together with an IS3 family transposase (Figure 3C). A high level of homology (>99%) was found between the *erm*(B) genes from *L. crispatus* CHCC3692 and *erm*(B) genes from *Streptococcus agalactiae*, *S. pyogenes*, *Enterococcus faecium*, and *L. reuteri*, indicating a common origin of the *erm*(B) genes of these Gram-positive bacterial species [36].

A *tet*(C) gene was identified on a metagenomic contig from the jejunum of swine #18 (Supplementary Tables S1 and S5, Figure 3D). This contig shared 99.6% identity in a 5830 bp region with that of the *tet*(C) genomic island of the *Chlamydia suis* strain R27, containing the *tet*(C) gene and the insertion sequence IS*cs605* encoding two predicted transposases *orfB* and *orfA* (NCBI Accession: AY428551 [37]). The *C. suis* insertion sequence IS*cs605* is the first insertion sequence identified in any chlamydia and belongs to the IS*605* family of insertion sequences [38]. *C. suis* has naturally acquired genes encoding for tetracycline resistance, where the architecture of the Tet-island was variable but the *tet*(C) gene was always intact, and recombination was a key factor in its transmission among *C. suis* isolates [39]. The *orfA* gene, which is related to members of the IS200 family of transposases, was suggested to be essential for both transposition activity and insertion specificity, whereas *orfB* within the IS element may have been important for the integration of the *tet*(C) islands in *C. suis* (Figure 3D [37]). Tetracycline-resistant *C. suis* strains were reported in pigs worldwide, including from China, Israel, Cyprus, the USA, and Western European countries such as Switzerland, Austria, Germany, Italy, and Belgium [39]).

### 2.4. Identification of Acquired ARGs in the Carp Intestinal Microbiome

The ARGs detected in the common carp intestinal microbiome are listed in Table 1 and Supplementary Tables S1 to S4 and a summary of the associated antibiotic classes in Figure 2B. Altogether, 12 different ARG types were detected, out of which four conferred resistance against the tetracyclines and three against β-lactams. Other antibiotic classes included aminoglycosides, quinolones, sulphonamides, and trimethoprim. Therefore, the tetracycline class contained the highest number of ARG types, followed by β-lactams and aminoglycosides. Of the identified ARGs, *tet*(E), an OXA-48 family β-lactamase gene, and *cphA4*, *ampS*, *aadA2*, *qnrS2*, *dfrA3*, and *sul1* were only identified in the carp intestine but not in the currently analyzed swine samples from Hungary. The identification of an OXA-48 family β-lactamase gene indicated that a carbapenemase enzyme was also present in the carp metagenome, conferring additional importance to the performed metagenomics data analyses.

### 2.5. Analysis of the Immediate Genetic Environment of ARGs on Selected Carp Intestinal Metagenomic Contigs

**Figure 4:**
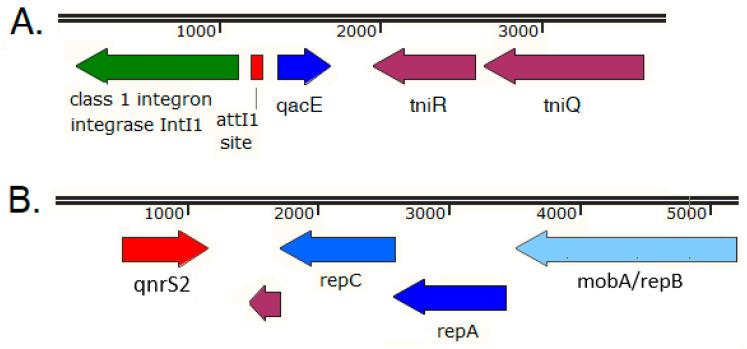
The immediate genetic environment of acquired resistance genes on selected carp intestinal metagenomics contigs. The 3754 bp contig carrying the *qacE* resistance determinant to quaternary ammonium compounds (**A**), and the 5852 bp carp contig harboring the *qnrS2* quinolone resistance determinant (**B**).

An Intl1 class 1 integrase gene was carried by a carp metagenomic contig of 3754 nucleotides (Figure 4A). A BLASTN search yielded a 3754 bp region of the commensal *Klebsiella pneumoniae* strain WM2b02 transposon Tn*1721* with the highest alignment score at 100% coverage and 99.9% identity (NCBI Accession HQ730118 [40]). The Intl1 integrase was followed by a full attI1 site and a single *qacE* gene cassette encoding a multidrug exporter that confers resistance to quaternary ammonium compounds, whereas the integron was negative for the 3′-Conserved Sequence (3′-CS) [40]. The detected full attI1 site is required for high efficiency recombinations with 59-be sites [41,42]. The examined 3754 bp carp metagenomic contig also carried the Tn*402*-like transposition genes *tniR* and *tniQ* (Figure 4A [40]).

A *qnrS2* quinolone resistance determinant was detected on a metagenomic contig of 5852 bp (Supplementary Table S4; Figure 4B) that was 99.9% identical in its full length with the corresponding regions of the following four plasmids: the *K. pneumoniae* strain I70 plasmid pKPSH70 (NCBI Accession KT896500), the *S. enterica* subsp. *enterica* serovar Cerro strain FSIS1607502 plasmid pF18S016-qnr (NCBI Accession CP082903), the *Aeromonas* sp. strain ZO2 plasmid pAerXIII (NCBI Accession MK962693), and the *Aeromonas* sp. strain 426 plasmid p426_p3 (NCBI Accession MT231821). These four IncQ plasmids are small in size (<8 kb) and include the plasmid mediated quinolone resistance determinant *qnrS*, the plasmid replication initiator *repC*, the helicase *repA* genes, and the *mobA-repB* (fusion gene of relaxase–primase) [43,44,45], whereas the associated *mobC* gene was not covered by the examined carp metagenomic contig (Figure 4B). It was suggested that these small *qnrS*-harboring pGNB2-like plasmids are ubiquitous in wastewater treatment facilities and are most likely of environmental origin [43].

Furthermore, an aminoglycoside adenyltransferase (*aadA2*) gene cassette of a class 1 integron was also identified on an 819 bp carp metagenomic contig (Supplementary Table S2), where *aadA2* was directly followed by the first 44 bp region of the 3′-Conserved Sequence (3′-CS) of a class 1 integron that was only partially covered by the contig. A *sul1* gene (Supplementary Table S4) was detected on a carp 1255 bp contig, were *sul1* is preceded by a partial *qacEΔ1* coding region (with its 5′ end not completely covered by the contig), indicating the presence of the 3′-CS of a class 1 integron [40]).

**Figure 5:**
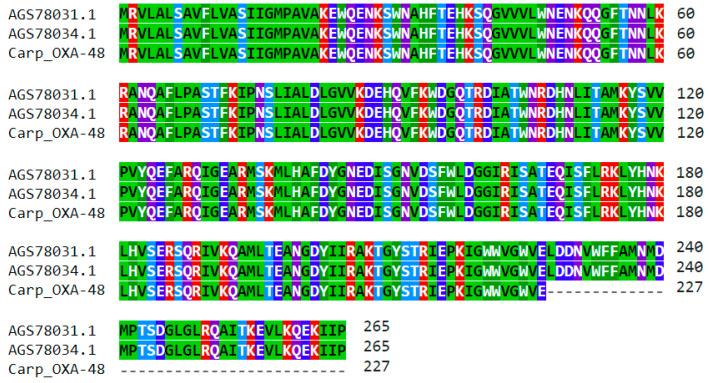
Multiple protein sequence alignment of the first 227 residues of an OXA-48 family class D β-lactamase encoded by the carp intestinal contig (Table 1) with the OXA-48b β-lactamases of *S. xiamenensis* strains IR24 and IR33, with NCBI Accessions AGS78031 and AGS78034 [46].

A 701 bp carp contig harbored a partial open reading frame encoding the first 227 residues of an OXA-48 family class D β-lactamase (85.4% coverage, Supplementary Table S3). The contig obtained the highest alignment score (at 100% identity) using a BLASTN search with the *bla*_OXA-48b_ carbapenemase genes of *Shewanella xiamenensis* strains IR24 and IR33 cultured from river water in Portugal (NCBI Accessions KC902850 and KC902851, [46]). The encoded protein sequence was 100% identical (in its first 227 amino acids covered by the contig) with the OXA-48b β-lactamases of *S. xiamenensis* strains IR24 and IR33 with NCBI Accessions AGS78031 and AGS78034, respectively (Figure 5).

Although only the partial coding sequence of the OXA-48 carbapenemase was present on the carp metagenomic contig, the first 227 encoded amino acids included residues 197–227 associated with the β5–β6 loop, which has a crucial role in the carbapenemase activity [47].

## 3. Discussion

Data collection on AMR (and the associated resistome) in food animals is required to enhance animal health measures, to promote the rational use of antimicrobials, and to identify specific therapeutic challenges related to AMR [48]. According to the third joint inter-agency report on the integrated analysis of antimicrobial consumption and resistance in humans and food-producing animals (that is, the third JIACRA report), bacterial resistance from humans was associated with that from food animals, where the latter was in turn related to antimicrobial consumption in animals. The most consistent positive association between AMR in food animals and between AMR from humans was found among *Campylobacter* spp. [4].

**Figure 6:**
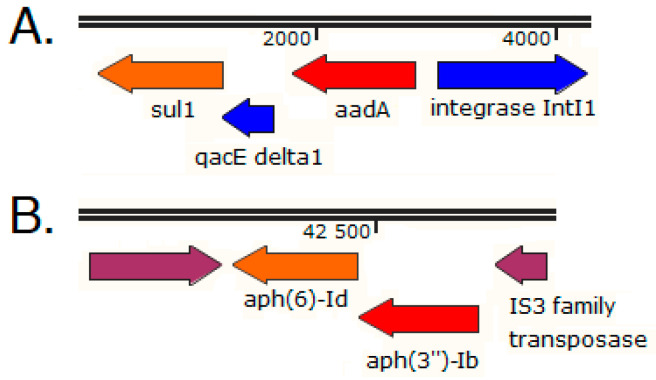
(**A**) Schematic structure of an *A. hydrophila* class 1 integron, as determined by PCR mapping experiments. IntI1: class 1 integrase, *aadA*: aminoglycoside-adenyltransferase, *qacEΔ1* and *sul1*: the 3′-Conserved Sequence (3′-CS) [77]. (**B**) Immediate genetic environment of the *aph(6)-Id* and *aph(3″)-Ib* genes carried by the *Moellerella wisconsensis* strain W1 plasmid pW1-a (NCBI Accession CP093256).

In addition, resistance genes carried by plasmids of commensal microbiota in food animals can spread between different animal hosts and humans [49]. Thus, the monitoring of AMR and the antibiotic resistance gene repertoire shall include both pathogens and commensal flora from healthy humans and animals and those with an infection [4]. Accordingly, a broad variety of mobile genetic elements involving various plasmids, transposons, and/or integrons was associated with the acquired ARGs discussed in this study, as indicated by their immediate genetic context (Figure 3, Figure 4, and Figure 6), suggesting a potential for horizontal transfer into other bacterial strains or species [50].

To the best of our knowledge, acquired ARGs have not yet been explored and reported from domestic pig and common carp intestinal samples from Hungary using shotgun metagenomic methods. The detected ARGs in most cases could not be unambiguously assigned to a specific bacterial genus or species, primarily because their immediate genetic environment often contained components of various MGEs (as exemplified by Figure 3 and Figure 4), and BLASTN searches with most of these metagenomic contigs yielded multiple hits with equal or very similar alignment scores from various bacterial genera or species, also pointing to the role of horizontal gene transfer in their dissemination.

Nonetheless, in certain cases such associations may be established with sufficient confidence, such as for linking the *cphA4* carbapenemase and the *ampS* β-lactamase genes detected in the carp intestinal microbiome with *Aeromonas* spp. [51,52], or the association of the *bla*_ACI-1_ β-lactamase gene (at 100% coverage and 100% identity) with the anaerobic coccus *Acidaminococcus intestinii* (*Acidaminococcaceae*) in the pig fecal microbiome [53,54] (Supplementary Table S3). Moreover, BLASTN searches of metagenomic contigs in some further cases also yielded database hits with both the coverage and identity at >99% and coupled with a specific bacterial genus for the highest alignment scores. Such observations suggested the association of a *tet*(Q) tetracycline resistance gene with *Bacteroides* spp. and a lincosamide nucleotidyltransferase *lnu*(C) determinant with *Megasphaera elsdenii* (*Veillonellaceae*) in the swine #2 duodenum and swine #18 fecal samples, respectively. These examples, together with the detailed metagenomic contig analyses described in Section 2.3 and Section 2.5, highlighted the advantages of the shotgun metagenomic approach in exploring the intestinal resistome of food animals as compared to characterizing isolates only from pre-selected species, such as *E. coli*, *S. enterica*, and/or *C. jejuni*.

A direct comparison of the diversity of acquired ARGs between the examined domestic pig versus carp intestinal samples (Figure 2) could not be established, as only one carp was sampled in the current study. Nonetheless, our data indicate a characteristically different ARG profile for the common carp raised in aquaculture: of the 12 ARG types detected in the carp intestinal microbiome eight genes (that is, 66%) were not demonstrated from the 15 intestinal samples analyzed from 12 domestic pigs (Table 1).

In total, 44 acquired ARG types were detected in the fecal samples of pigs, whereas a lower number, in total 38 ARG types, was detected in the pig small intestine, as summarized in Table 2. These results are similar to those reported by Hu et al. [55], who found that the overall absolute abundance of ARG families was higher in the colon of weaned piglets compared to their overall abundance in the ileum, including higher abundances in the colon for the tetracycline resistance ribosomal protection proteins, *erm*-type 23S ribosomal RNA methyltransferases, and others [55]. Within the swine intestinal tract there is a significantly higher microbial diversity and a higher concentration of bacteria in the colon compared to the ileum, and in the ileum compared to the proximal small intestine [56], which might have contributed to the higher diversity of ARG types detected in colon and fecal samples.

Most of the acquired ARGs detected by metagenomic methods in domestic pig gut microbiota in the current study (Table 1) were not reported in Hungary by earlier studies applying alternative molecular approaches, such as those to characterize porcine *E. coli* isolates from Hungary [57,58], with the exceptions of *tet*(A), *tet*(B), *aadA1*, *strA*, *strB*, and *sul2*. Meanwhile, among the acquired ARGs detected in the swine intestinal microbiome in Hungary, several determinants, including *mef*(A), *lnu*(B), *lnu*(C), *erm*(B), *tet*(L), *tet*(O), *tet*(Q), *tet*(W), *tet*(40), *tet*(44), *tetA*(P), *CatP*, and others, were also detected in a cross-sectional metagenomic study involving 176 conventional pig farms in nine European countries [31]. Of these ARGs, *tet*(Q), *tet*(W), *tet*(O), and *mef*(A) were also present in fecal samples of free-living wild boars from Füzérkomlós in the Zemplén Mountains in Hungary (Section 4.1) [22]. Interestingly, *tet*(Q), *tet*(W), and *tet*(O) were overall the first, second, and fourth most abundant tetracycline resistance genes, respectively, within their antimicrobial class at the examined 176 European domestic pig farms, and *mef*(A) was overall the most abundant macrolide resistance gene within its antimicrobial class [31]. In line with their reported high abundances on European pig farms, the *tet*(Q), *tet*(W), and *mef*(A) genes were demonstrated in all four domestic pig intestinal segments examined in this study (see Section 2.2). Our data and observations also suggest that the occurrence of certain antibiotic resistance genes in the gut microbiome of free-living wild animals may correlate with their prevalence in food-producing farm animal populations [22].

Within the overall sales of antimicrobials for food-producing animals in 31 European countries the largest amounts were constituted by penicillins (31.1%), tetracyclines (26.7%), and sulfonamides (9.9%) in 2020, with 2019 and 2020 being the first years in which the overall proportion of penicillin sales was higher than that of tetracyclines. However, in 2020 in Hungary the sales for tetracyclines (57.5 mg/Population Correction Unit/PCU/) still preceded those of penicillins (51.6 mg/PCU) [8]. Data on tetracycline sales were consistent with the high variety and proportion of acquired tetracycline resistance genes detected in Hungary in domestic pig and carp intestinal specimens (Figure 2C). Associations were previously reported between the relative abundances of *erm*(B) and lincosamide and macrolide use, and between total antimicrobial use during the fattening phase of pigs and the relative abundances of *aph(3′)-III* and *sul2* [14].

Considering the taxonomic annotations of metagenomic contig sets summarized in Figure 1, the markedly higher proportions of *Mycoplasmataceae* contigs obtained for the pig small intestine compared to the fecal samples reflect a similar pattern to that reported by Liu et al. [59], where *Mycoplasma* was detected as a dominant genus only in the duodenum, jejunum, and ileum, but not in the feces of piglets fed a corn- and soybean-meal-based diet. However, the relative abundance of *Mycoplasma* in the jejunum mucosal microbiota decreased significantly when their diet was partly replaced with 5% alfalfa meal [59]. Furthermore, changes of porcine gut microbiota in response to dietary chlorogenic acid supplementation (CGA, one of the most abundant dietary polyphenols present in a variety of foods and beverages) revealed that pigs fed a CGA-supplemented diet tended to (0.05 < *p* < 0.10) have an increased abundance of *Mycoplasma* in their cecal content samples [60]. These examples illustrate that *Mycoplasma* may display substantial relative abundances in piglet intestinal sections in response to various dietary effects that were not within the scope of the present analyses.

The acquired resistome of the fish intestinal microbiome has not yet been examined in Hungary by metagenomic methods; however, the acquired ARGs identified in the common carp microbiome in Hungary, including *qnrS2* and *bla*_OXA-48_-like genes, have been reported in fish by earlier international metagenomic studies, too [15,61,62,63].

OXA-48-producing *K. pneumoniae* was isolated from four patients in four Western Hungarian medical centers in 2014 [64]. Furthermore, *bla*_OXA-48_-like genes were also demonstrated in six Enterobacterales human clinical isolates cultured in the period of 2014–2017 in Szeged, Southern Hungary [65], from the same geographical region where the carp intestinal contig harboring an OXA-48 family β-lactamase gene was detected (Supplementary Table S3). OXA-type carbapenemases have not yet been reported from livestock samples in Hungary. The carbapenem-hydrolyzing class D β-lactamases of the OXA-48-type are of great concern because of their rapid worldwide spread and their propensity to evolve by mutations, leading to various phenotypic characteristics, wherein OXA-48-type enzymes have so far been identified only in the Enterobacterales [66]. Over the past two decades, OXA-48-like enzymes have become the most prevalent carbapenemases among the *Enterobacteriaceae* across much of Europe, North Africa, and the Middle East. These types of ARGs are difficult to detect by phenotypic methods because they often cause only low level in vitro resistance to carbapenems [67]. Carbapenems are not licensed for use in veterinary medicine in the EU; nevertheless, carbapenemase-producing *Enterobacteriaceae* isolates have been sporadically detected in livestock, companion animals, and wildlife in recent years [68]. Earlier reports of OXA-48-producing human clinical isolates from Hungary suggest that the OXA-type carbapenemase was possibly introduced into aquaculture through colonized human personnel, or alternatively, from a yet unknown environmental source. Therefore, future studies conducted by a comprehensive One Health approach are required to explore the epidemiology and spread of such high risk acquired ARGs between the healthcare settings, the food chain, and their potential environmental reservoirs [19].

Plasmid mediated quinolone resistance was explored among porcine *E. coli* isolated in 2008 from two large pig farms in Romania and Hungary [69], and *qnrS1*-positive strains were identified from the Romanian pig farm, but not from the Hungarian farm. On the other hand, the testing of 237 ESBL-producing *Enterobacteriaceae* isolates obtained from seven different hospitals and clinics between 2002 and 2006 in Hungary for plasmid mediated quinolone resistance determinants revealed that their prevalence was 3% for *qnrA*, 0.8% for *qnrB*, and 0.4% for *qnrS*, where *K. pneumoniae* contained most of these plasmid-borne ARGs [70]. Among 103 ESBL-producing *Enterobacteriaceae* isolates from bloodstream infections treated at the intensive care units of Semmelweis University in Budapest between 2010 and 2014, six strains (one *E. coli*, four *K. pneumoniae*, and one *K. oxytoca*) proved to be *qnrS*-positive by PCR, but the specific *qnrS* allele was not determined [71]. In light of these earlier findings, the description of a *qnrS2* quinolone resistance determinant on a carp metagenomic contig from Hungary (Supplementary Table S4; Figure 4B) signifies the first report of *qnrS* (and its *qnrS2* allele) from a food-producing animal in Hungary and also demonstrates the occurrence of *qnrS* genes in bacterial strains of human, animal, and wastewater origin in Hungary (Supplementary Table S4, [72]).

Plasmid mediated quinolone resistance was first identified in a *K. pneumoniae* clinical isolate from the United States in 1998 [73]. Since then, the wide dissemination of *qnr* genes has been reported in both clinical and environmental strains, and the *qnrS2* genes belong to the most prevalent plasmid mediated quinolone resistance determinant group, identified mainly in representatives of *Enterobacteriaceae* (*E. coli*, *S. enterica* sv. Typhimurium, *Klebsiella* spp.) but also in *Shewanella* sp., *Vibrionaceae*, and *Aeromonas* spp. [44]. The *qnrS2* gene was also reported in *Aeromonas punctata* subsp. *punctata* and *A. media* recovered from the river Seine at two sampling sites in East and South Paris. Its identification in *Aeromonas* spp. strains highlighted the role of water as a vehicle for the dissemination of the *qnrS*-resistance determinants [74], similar to the environment of fish production in aquaculture. Indeed, two of the ARGs that were detected only in the carp intestine in the current study, *cphA4* and *ampS*, are characteristic ARGs of *Aeromonas* spp., an important genus for fish-pathogenic bacteria [52].

An untreated sewage wastewater sample from the North Pest Wastewater Treatment Plant (Budapest, Hungary) was collected in 2016 and was subjected to shotgun metagenomic sequencing as part of a global study to monitor antimicrobial resistance genes in urban sewage [72]. A comparative analysis showed that 10/15 (66.6%) tetracycline resistance genes, 6/16 (37.5%) aminoglycoside resistance genes, 4/10 (40.0%) β-lactamases, and 10/18 (55.5%) other ARGs identified in the current study from the intestinal microbiome of food animals were also detected in urban wastewater in Hungary (Supplementary Tables S1–S4), where the North Budapest plant treats both communal wastewater and organic wastes of animal origin. The direct agricultural use of sewage sludge (that is, sludge applied on agricultural land) was in the range of about 24,000–42,000 metric tons per year in Hungary between 1998 and 2011 [75]. The ARGs entering wastewater treatment plants from communal wastewater or from animal organic wastes can further disseminate onto agricultural lands through sewage sludge application [76].

Indeed, a gentamicin-resistant *A. hydrophila* strain carrying a class 1 integron-borne aminoglycoside-adenyltransferase *aadA* gene was cultured from sewage sludge of the South Pest Wastewater Treatment Plant in Budapest (Figure 6A) [77]. As sewage sludge is a byproduct of wastewater treatment, this observation indicated that the wastewater treatment process did not eliminate viable resistant bacteria from the resulting sludge. Integrons with this identified structure have also been reported from various clinical, environmental, and fish pathogenic *Aeromonas* spp. strains, often indicating the presence of broad-host-range resistance plasmids of the IncU group [77,78,79,80,81].

A 1149 bp metagenomic contig from the North Pest Wastewater Treatment Plant in Budapest harboring *aph(6)-Id* (*strB*) and *aph(3″)-Ib* (*strA*) aminoglycoside resistance genes showed 100% coverage and 99.9% identity (1148/1149 bp) with the corresponding region of the *Moellerella wisconsensis* (*Enterobacteriaceae*) strain W1 plasmid pW1-a cultured from red deer meat produced in Hungary (NCBI Accession CP093256, Figure 6B). Furthermore, *aadA13* and/or *aadB* aminoglycoside resistance genes identified from the North Pest Wastewater Treatment Plant [72] were carried by ST175 and ST395 *Pseudomonas aeruginosa* clones with a countrywide distribution in Hungary, recovered from 13 hospitals from 11 towns, including three hospitals in Budapest [82]. The above observations and others [83,84] underscore the need for a One Health approach to thoroughly explore the possible role of environmental reservoirs in the epidemiology and spread of antimicrobial-resistance determinants between different hosts and habitats.

In conclusion, by use of an up-to-date metagenomic approach, a broad variety of acquired antimicrobial resistance determinants and associated MGEs were demonstrated in the microbiome of domestic pig and common carp intestinal samples from Hungary, including ARGs for two critically important antimicrobial classes, quinolones and carbapenems. These ARGs may disseminate to further agricultural and natural environments through feces/manure/sludge or through the water systems, and potentially also to the human population through the food production chain. It was suggested that because of the high microbial loads and resistome diversity found on farms, the meat production chain may provide an important means of transmission of antibiotic resistance genes to humans, mainly through bacteria colonizing animal surfaces during the evisceration and dressing processes [85]. The detection of the same ARGs in the gut microbiome of domestic pigs in Hungary that were earlier already identified from free-living wild boars highlights a possible correlation between the acquired resistome of domestic and wild animal populations. Monitoring and understanding the distribution and means of dissemination for antimicrobial resistance determinants by a comprehensive One Health approach is therefore necessary for both national and European mitigation strategies. The use of NGS technologies (such as shotgun metagenomic sequencing) may extend the range of detected ARGs in complex bacterial communities compared to, for example, conventional PCR-based methods, thus supporting more comprehensive data collection and subsequent epidemiological analysis.

## 4. Materials and Methods

### 4.1. Sample Collection and Processing

**Figure 7:**
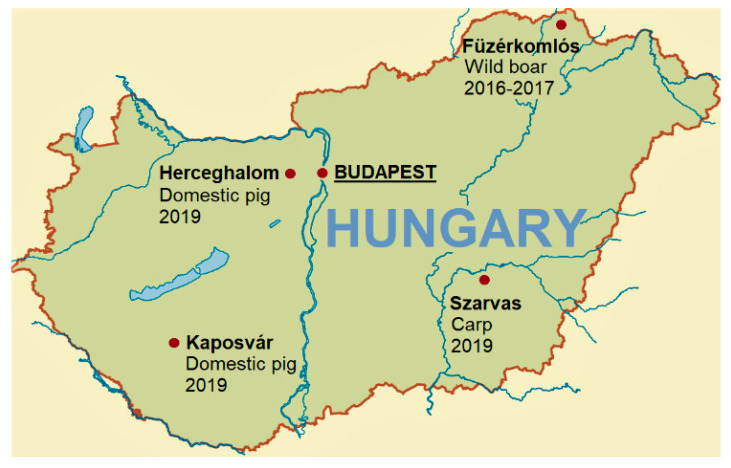
Geographical locations of the sampling sites discussed in this study. Budapest, the capitol, is indicated by underlined letters. The wild boar intestinal content samples collected near Füzérkomlós were examined by shotgun metagenomic sequencing and analyses in a previous study [22].

About 1 to 5 g of intestinal content samples from the duodenum, jejunum, and ileum (the small intestine) and from feces were collected for analyses of eleven 10-week-old weaned male Danbred piglets (*Sus scrofa*) averaging about 13.5 kg body weight in Kaposvár, Somogy County, in April 2019, as summarized in Supplementary Table S5. In addition, a fecal sample was collected from a 26-week-old female domestic pig of about 120 kg in November 2019 in Herceghalom, Pest County. In order to compare the intestinal resistomes between domestic pigs and an important aquaculture species from Hungary, the intestinal content was also sampled from a 20-week-old common carp (*Cyprinus carpio*) with a weight of 102 g and a total length of 17.5 cm (sample code P3F, Supplementary Table S5). The carp sampling was performed in October 2019 in Szarvas (Békés County, Figure 7). Intestinal content samples (from the ileum, colon, and rectum) were collected and analyzed by metagenomic methods in a previous study [22] from five free-living wild boars (*Sus scrofa*) shot during the regular hunting season in November 2016 and January 2017 near Füzérkomlós, in the Zemplén Mountains in Northeast Hungary (Figure 7).

### 4.2. DNA Purification and Metagenomic Sequencing

The collected intestinal content samples were evenly homogenized by mixing them using a sterile metal spoon. DNA was purified from about 0.1 g of the carp P3F intestinal and swine S1F fecal samples (Supplementary Table S5) by Xenovea Ltd. (Szeged, Hungary) using the AquaGenomic Kit (MultiTarget Pharmaceuticals, Salt Lake City, UT, USA) following the instructions from the manufacturer. Library preparation was attained by enzymatic sheering with solid phase reversible immobilization (SPRI) size selection using the KAPA HyperPlus Kit and KAPA HyperPure Beads (Roche Sequencing Solutions, Inc., Pleasanton, CA, USA). Barcoded adapter ligation and post-ligation amplification were followed by quality control and shotgun metagenomic sequencing on the Illumina NextSeq 500 platform (Illumina Inc., San Diego, CA, USA) using 2 × 150 bp paired-end chemistry by Xenovea Ltd. (Szeged, Hungary).

For the domestic pig intestinal content samples collected in Kaposvár (Somogy County), DNA purification was performed by IMGM Laboratories GmbH (Martinsried, Germany) using the NukEx Pure RNA/DNA Kit (gerbion GmbH & Co. KG, Kornwestheim, Germany). Samples were subjected to bead beating with 0.4–0.6 mm zirconia beads for 2 min prior to DNA isolation. For quality control, gDNA concentrations were quantified using the QubitTM dsDNA HS Assay Kit (Thermo Fisher Scientific, Waltham, MA, USA). The NEBNext UltraTM II FS DNA Library Preparation for Illumina Sequencing Kit (New England Biolabs, Ipswich, MA, USA) was applied to generate metagenomic sequencing libraries from the gDNA samples according to the manufacturer’s protocol. Library preparation was performed with 100 ng gDNA as input and 8 PCR cycles. The quality and fragment distribution of the NEBNext DNA UltraTM II libraries were analyzed using the High Sensitivity DNA Kit (Agilent Technologies Inc., Santa Clara, CA, USA) on a 2100 Bioanalyzer (Agilent Technologies, Santa Clara, CA, USA). The library was sequenced at a final concentration of 450 pM and with a 1% PhiX v3 control library spike-in (Illumina Inc., San Diego, CA, USA) on the NovaSeq 6000 sequencing system (Illumina Inc., San Diego, CA, USA). For cluster generation and sequencing, NovaSeq 6000 S1 v1.5 flow cells with 300 cycles (2 × 150 bp) were run. Sequencing was operated under the control of the NovaSeq Control Software v 1.7.0 (Illumina Inc., San Diego, CA, USA).

The FastQC Version 0.11.9 tool (Babraham Bioinformatics, Cambridge, UK) was used for analyzing the quality and quantity of the raw NGS data before metagenomic contig assembly and functional analyses. FastQC analysis showed that the most frequently observed mean per sequence quality scores were >34 for both the forward and reverse Illumina reads, with 0% per base ambiguous nucleotide (N) content, indicating high quality base calling for the obtained 2 × 150 bp NGS reads.

### 4.3. Metagenomic Contig Assembly

Metagenomic contig assembly was performed on the Galaxy platform [86] by the MEGAHIT version 1.2.9 NGS de novo assembler, designed for assembling large and complex metagenomics data. MEGAHIT assembles the data as a whole, with no pre-processing like partitioning or normalization required [87]. For contig assembly 12.7–60.4 million paired-end sequencing reads per sample were used. Basic statistical analysis and summary of the obtained metagenomic contigs were performed using the Fasta Statistics Galaxy version 1.0.0 tool [88]; see Supplementary Table S6. Metagenomic contigs were also assembled and analyzed from shotgun metagenomic sequencing reads for untreated wastewater collected in 2016 from the North Pest Wastewater Treatment Plant (Budapest, Hungary) with the NCBI SRA code ERR1713361 [72].

### 4.4. Identification of ARGs Harbored by the Metagenomic Contigs

The ABRicate Galaxy version 1.0.1 tool [32,89,90,91,92] was applied with the ResFinder 4.1 database [93] for the identification of acquired ARGs harbored by the metagenomic contigs, with the settings of a ≥95% threshold for sequence identity [90,92,94,95] and a minimum coverage of ≥60% [26,91,93]. ResFinder is aimed at detecting ARGs that are involved in acquired antibiotic resistance [93]. Matrices of detected ARGs were generated using the ABRicate tool. Methods and analyses applied in this study for metagenomic contig assembly and ARG detection were validated as described earlier [22].

### 4.5. Annotation and Functional Analysis of Selected Metagenomic Contigs

Selected metagenomic contigs of sufficient size and harboring ARGs were annotated using the Prokka Prokaryotic genome annotation Galaxy version 1.14.6 tool [96] and by individual BLASTP searches of translated open reading frames against the NCBI Protein databases. Schematic diagrams of the annotated contigs were generated by SnapGene Viewer v 3.2.1 (Insightful Science, Woburn, MA, USA).

### 4.6. Taxonomic Classification of the Assembled Metagenomic Contigs

The relative proportions of the assembled metagenomic contigs assigned to different bacterial families were assessed by the Kraken Galaxy version 1.3.1 tool [97] using the standard Kraken “Bacteria” database that contains the complete NCBI taxonomic information and the complete genomes in the NCBI RefSeq Reference Sequence Database for the bacterial domain [97]. Data presented in Figure 1 summarize the taxonomic annotations of the assembled metagenomic contigs to various bacterial families, rather than the bacterial community composition in the analyzed intestinal segments [98,99]. This is partly because a fraction of the metagenomic sequencing reads cannot be assembled to metagenomic contigs, especially those from low abundance species, and this fraction of non-assembled reads depends on several factors, such as sequencing depth and microbiome diversity [100]. Furthermore, the analyzed individual metagenomic contigs had a wide range of contig lengths (Supplementary Table S6), and thus represent diverse numbers of Illumina sequencing reads assembled by MEGAHIT.

## Figures and Tables

**Table 1:** Summary of the ARG types detected in domestic pig and common carp intestinal samples from Hungary ^a^.

	Aminoglycoside	Tetracycline	β-Lactam	Macrolide	Others
Domestic pig	*aac(6′)-Im* *aac(6′)-aph(2″)* *ant(3″)-Ia (aadA1)* *ant(6)-Ia (aadE)* *ant(6)-Ib* *ant(9)-Ia* *aph(2″)-Ib* *aph(2″)-Ic* *aph(2″)-If* *aph(2″)-Ih* *aph(3′)-Ia* *aph(3″)-Ib (strA)* *aph(6)-Id (strB)* *aph(3′)-III* *rmtF*	*tet*(40) *tet*(44) *tet*(A) *tet*(B) *tet*(C) *tet*(H) *tet*(L) *tet*(M) *tet*(O) *tet*(Q) *tet*(W) *tet*(X) *tetA*(P) *tetB*(P)	*bla*_ACI-1_ OXA-61 family β-lactamase *bla*_ROB-1_ *cfxA3* *cfxA4* *cfxA5* *cfxA6*	*lnu*(B) *lnu*(C) *lnu*(P) *lsa*(E) *mef*(A) *msr*(D) *erm*(B) *erm*(F) *erm*(G) *erm*(Q) *vatE*	*catP* *cfr*(C) *nimJ* *sul2*
Common carp	*ant(3″)-Ia (aadA2)* *aph(3″)-Ib (strA*)	*tet*(A) *tet*(B) *tet*(E) *tet*(X)	*ampS* *cphA4* OXA-48-type carbapenemase		*dfrA3* *qnrS2* *sul1*

^a^ ResFinder antibiotic classes were applied according to Munk et al. (2018) and Van Gompel et al. (2019) [30,31].

**Table 2:** The number and proportion (%) of ARG types of different antibiotic classes detected in swine small intestinal and fecal samples.

	Aminoglycosides		Tetracyclines		β-Lactams		Other Classes		In Total	
	No. ^c^	(%)	No.	(%)	No.	(%)	No.	(%)	No.	(%)
Small intestine	11	21.57	12	23.53	3	5.88	12	23.53	38	74.50
Small intestine only ^a^	2	3.92	2	3.92	1	1.96	1	1.96	6	11.76
Fecal samples	13	25.49	11	21.57	6	11.76	14	27.45	44	86.27
Fecal samples only ^b^	4	7.84	2	3.92	4	7.84	2	3.92	12	23.52
In total	15	29.41	14	27.45	7	13.73	15	29.41	51	100

^a^ ARG types detected only in duodenum, jejunum, and/or ileum, but not in feces; ^b^ ARG types detected only in feces, but not in duodenum, jejunum, and/or ileum; ^c^ No.: the number of ARG types detected.

## Data Availability

The raw NGS-sequencing reads analyzed in this study were deposited in the National Center for Biotechnology Information Sequence Read Archive (NCBI SRA) database with the BioProject Accession PRJNA823519.

## Supplementary Materials

The following supporting information can be downloaded at: https://www.mdpi.com/article/10.3390/antibiotics11101441/s1, Table S1. Acquired tetracycline resistance determinants identified in domestic pig and common carp intestinal microbiomes from Hungary; Table S2. Acquired aminoglycoside resistance determinants identified in domestic pig and common carp intestinal microbiomes from Hungary; Table S3. Acquired β-lactame resistance determinants identified in domestic pig and common carp intestinal microbiomes from Hungary; Table S4. Acquired resistance determinants of other antibiotic classes identified in domestic pig and common carp intestinal microbiomes from Hungary. Table S5. Metadata for the intestinal samples from Hungary discussed in this study; Table S6. Basic statistics for the metagenomic contigs.
