# Supplementary material for: Detection of Acquired Antibiotic Resistance Genes in Domestic Pig (Sus scrofa) and Common Carp (Cyprinus carpio) Intestinal Samples by Metagenomics Analyses in Hungary
Source: Antibiotics (Basel). 2022 Oct 20;11(10):1441. doi: 10.3390/antibiotics11101441 (PMC9598914; doi:10.3390/antibiotics11101441)
Supplement: Supplementary file 1 [file antibiotics-11-01441-s001.zip › antibiotics-1975417-supplementary.pdf]

Supplementary Table S1. Acquired tetracycline resistance determinants identified in domestic pig and common carp intestinal microbiomes from Hungary

Resistance Determinant	Resistance Mechanism	Highest Coverage in the Different Sample Types (%)				
		Domestic Pig, Duodenum	Domestic Pig, Jejunum	Domestic Pig, Ileum	Domestic Pig, Faeces	Common Carp, Intestine
tet(40)	major facilitator superfamily (MFS) antibiotic efflux pump	100.00	.	100.00	100.00	.
tet(44) [a]	ribosomal protection protein	.	.	100.00	96.98	.
tet(A)	major facilitator superfamily (MFS) antibiotic efflux pump	.	.	.	72.00	97.80
tet(B)	major facilitator superfamily (MFS) antibiotic efflux pump	.	100.00	100.00	.	70.65
tet(C)	major facilitator superfamily (MFS) antibiotic efflux pump	.	100.00	.	100.00	.
tet(E)	major facilitator superfamily (MFS) antibiotic efflux pump	.	.	.	.	100.00
tet(H)	major facilitator superfamily (MFS) antibiotic efflux pump	.	.	98.56	.	.
tet(L)	major facilitator superfamily (MFS) antibiotic efflux pump	.	99.93	.	99.93	.
tet(M)	ribosomal protection protein	.	100.00	84.48	100.00	.
tet(O)	ribosomal protection protein		.	100.00	70.99	.
tet(Q)	ribosomal protection protein	100.00	100.00	100.00	90.97	.
tet(W)	ribosomal protection protein	100.00	99.69	94.84	.	
tet(X)	flavin-dependent monooxygenase	.	.		100.00	100.00
tetA(P)	major facilitator superfamily (MFS) antibiotic efflux pump	.	96.20	96.36	96.36	.
tetB(P)	ribosomal protection protein	.	100.00	100.00	100.00	.

[a] Bold blue charaters indicate a resistance determinant also identified in untreated wastewater from the Budapest North Wastewater Treatment Plant in Hungary by Hendriksen et al. (2019) [72]. NCBI BioSample: SAMEA4527629

Supplementary Table S2. Acquired aminoglycoside resistance determinants identified in domestic pig and common carp intestinal microbiomes from Hungary

Resistance Determinant	Resistance Mechanism	Highest Coverage in the Different Sample Types (%)				
		Domestic Pig, Duodenum	Domestic Pig, Jejunum	Domestic Pig, Ileum	Domestic Pig, Faeces	Common Carp, Intestine
aac(6')-Im	aminoglycoside acetyltransferase		.	100.00	100.00	.
aac(6')-aph(2'') [a]	aminoglycoside acetyl and phosphotransferase		100.00	.	70.69	.
ant(3'')-Ia (aadA1) [b]	aminoglycoside nucleotidyltransferase	.	.		100.00	
ant(3'')-Ia (aadA2)	aminoglycoside nucleotidyltransferase		.		.	90.15
ant(6)-Ia (aadE)	aminoglycoside nucleotidyltransferase	100.00	100.00	100.00	100.00	.
ant(6)-Ib	aminoglycoside nucleotidyltransferase	.	.	100.00	100.00	.
ant(9)-Ia	aminoglycoside nucleotidyltransferase	.	.	.	69.48	.
aph(2'')-Ib	aminoglycoside phosphotransferase	.	.	100.00	100.00	.
aph(2'')-Ic	aminoglycoside phosphotransferase	.	100.00	.	100.00	.
aph(2'')-If	aminoglycoside phosphotransferase	100.00	100.00	100.00	100.00	.
aph(2'')-Ih	aminoglycoside phosphotransferase	.	.	.	100.00	.
aph(3')-Ia	aminoglycoside phosphotransferase	.	.	100.00	.	.
aph(3'')-Ib (strA)	aminoglycoside phosphotransferase	.	99.88	99.88	72.14	66.55
aph(6)-Id (strB)	aminoglycoside phosphotransferase	.	.	76.70	.	
aph(3')-III	aminoglycoside phosphotransferase	.	100.00	100.00	100.00	.
rmtF	16S rRNA methyltransferase	.	.	.	100.00	.

[a] Bold blue charaters indicate a resistance determinant also identified in untreated wastewater from the Budapest North Wastewater Treatment Plant in Hungary by Hendriksen et al. (2019) [72]. NCBI BioSample: SAMEA4527629

[b] *aadA1* is included in the aadA_clust1 ResFinder gene cluster as described by Munk et al. (2018) and Hendriksen et al., (2019) [30, 72]

Supplementary Table S3. Acquired β-lactame resistance determinants identified in domestic pig and common carp intestinal microbiomes from Hungary

Resistance Determinant	Resistance Mechanism	Highest Coverage in Different Sample Types (%)				
		Domestic Pig, Duodenum	Domestic Pig, Jejunum	Domestic Pig, Ileum	Domestic Pig, Faeces	Common Carp, Intestine
bla $_{ACI-1}$	Ambler class A extended-spectrum β-lactamase	100.00	100.00	63.05	100.00	-
bla $_{OXA}$	Ambler class D OXA-61 family β-lactamase	-	-	-	99.35	-
bla $_{OXA}$	Ambler class D OXA-48 family carbapenem-hydrolyzing β-lactamase	-	-	-	-	85.46
ampS (*bla* $_{OXA}$)	Ambler class D OXA-12 family β-lactamase	-	-	-	-	97.23
bla $_{ROB-1}$	Ambler class A broad spectrum β-lactamase	-	100.00	100.00	-	-
cfxA3 [a,b]	Ambler class A β-lactamase	-	-	75.47	100.00	-
cfxA4 [a,b]	Ambler class A β-lactamase	-	-	-	100.00	-
cfxA5 [a,b]	Ambler class A β-lactamase	-	-	-	100.00	-
cfxA6 [a]	Ambler class A β-lactamase	-	-	-	100.00	-
cphA4	Ambler class B, subclass B2 metallo-β-lactamase, carbapenemase	-	-	-	-	99.48

[a] Bold blue charaters indicate a resistance determinant also identified in untreated wastewater from the Budapest North Wastewater Treatment Plant in Hungary by Hendriksen et al. (2019) [72]. NCBI BioSample: SAMEA4527629

[b] included in the cfxA_clust ResFinder gene cluster as described by Munk et al. (2018) and Hendriksen et al., (2019) [30, 72]

Supplementary Table S4. Acquired resistance determinants of other antibiotic classes identified in domestic pig and common carp intestinal microbiomes from Hungary

Antibiotic Class	Resistance Determinant	Resistance Mechanism	Highest Coverage in the Different Sample Types (%)				
			Domestic Pig, Duodenum	Domestic Pig, Jejunum	Domestic Pig, Ileum	Domestic Pig, Faeces	Common Carp, Intestine
Phenicol	*catP*	chloramphenicol acetyltransferase	.	.	.	100.00	.
Phenicol	*cfrC*	23S rRNA methyltransferase	.	.	67.23	100.00	.
Trimethoprim	*dfrA3*	dihydrofolate reductase	.	.	.	.	61.76
Macrolide	***mef(A)*** [a]	macrolide efflux protein A	100.00	100.00	100.00	100.00	.
Macrolide	***msr(D)***	ribosomal protection protein	.	.		100.00	.
Macrolide	***erm(B)***	23S ribosomal RNA methyltransferase	100.00	100.00	.	100.00	.
Macrolide	***erm(F)***	23S ribosomal RNA methyltransferase	.	99.88	.	100.00	.
Macrolide	***erm(G)***	23S ribosomal RNA methyltransferase	.	100.00	100.00	100.00	.
Macrolide	*erm(Q)*	23S ribosomal RNA methyltransferase	.	100.00	100.00	100.00	.
Macrolide	***lnu(B)***	lincosamide nucleotidyltransferase	.	100.00	100.00	100.00	.
Macrolide	***lnu(C)***	lincosamide nucleotidyltransferase	100.00	100.00	100.00	100.00	.
Macrolide	*lnu(P)*	lincosamide nucleotidyltransferase	.	100	100	100.00	.
Macrolide	*lsaE*	ribosomal protection protein	.	100.00	100.00	100.00	.
Macrolide	*vat(E)*	streptogramin acetyltransferase	.	.	.	66.98	.
Nitroimidazole	*nimJ*	nitroimidazole reductase gene	77.71	.	.	100.00	.
Quinolone	***qnrS2*** [b]	antibiotic target protection	.	.	.	.	100.00
Sulphonamide	***sul1*** [c]	dihydropteroate synthase	.	.	.	.	100.00
Sulphonamide	***sul2*** [d]	dihydropteroate synthase	78.51	100.00	100.00	.	.

[a] Bold blue charaters indicate a resistance determinant also identified in untreated wastewater from the Budapest North Wastewater Treatment Plant in Hungary by Hendriksen et al. (2019) [72]. NCBI BioSample: SAMEA4527629

[b] *qnrS2* is included in the QnrS_clust2 ResFinder gene cluster as described by Munk et al. (2018) and Hendriksen et al., (2019) [30, 72]

[c] *sul1* is included in the sul1_sul3_clust ResFinder gene cluster as described by Munk et al. (2018) and Hendriksen et al., (2019) [30, 72]

[d] *sul2* is included in the sul2_clust ResFinder gene cluster as described by Munk et al. (2018) and Hendriksen et al., (2019) [30, 72]

Supplementary Table S5. Metadata for the intestinal content samples from Hungary discussed in this study									
Species	Age	Location, County	Time of sampling	Code of animal	Code of intestinal sample				References
					Duodenum	Jejunum	Ileum	Faeces	
Sus scrofa (domestic pig)	10 weeks	Kaposvár, Somogy	April 2019	2	2d	2j		F212	this work
Sus scrofa (domestic pig)	10 weeks	Kaposvár, Somogy	April 2019	4		4j			this work
Sus scrofa (domestic pig)	10 weeks	Kaposvár, Somogy	April 2019	7	7d				this work
Sus scrofa (domestic pig)	10 weeks	Kaposvár, Somogy	April 2019	11				F2111	this work
Sus scrofa (domestic pig)	10 weeks	Kaposvár, Somogy	April 2019	12			12i		this work
Sus scrofa (domestic pig)	10 weeks	Kaposvár, Somogy	April 2019	14			14i		this work
Sus scrofa (domestic pig)	10 weeks	Kaposvár, Somogy	April 2019	15				F2115	this work
Sus scrofa (domestic pig)	10 weeks	Kaposvár, Somogy	April 2019	16			16i		this work
Sus scrofa (domestic pig)	10 weeks	Kaposvár, Somogy	April 2019	17				F2117	this work
Sus scrofa (domestic pig)	10 weeks	Kaposvár, Somogy	April 2019	18		18j		F2118	this work
Sus scrofa (domestic pig)	10 weeks	Kaposvár, Somogy	April 2019	20				F2120	this work
Sus scrofa (domestic pig)	26 weeks	Herceghalom, Pest	November 2019	S1				S1F	this work
Cyprinus carpio (common carp)	20 weeks	Szarvas, Békés	October 2019	P3				P3F	this work

Supplementary Table S6. Basic statistics of the metagenomic contigs obtained for the domestic pig and common carp intestinal samples

Sample code	2d	7d	2j	4j	18j	12i	14i	16i	F212	F2111	F2115	F2117	F2118	F2120	S1F	P3F
Contig L50[a]	615068	686506	536157	694272	126065	125767	655429	645879	100607	95927	115243	88216	90407	102301	128811	98278
Contig N50[b]	669	512	512	612	535	464	767	780	1238	1300	1253	1376	1320	1268	631	667
Contig L90[c]	1636613	1679349	1290392	1799988	376702	367310	1808474	1780054	638115	544364	633606	531894	544674	599110	459535	351090
Contig N90[d]	376	341	341	356	332	322	400	401	365	382	383	388	380	376	334	339
Contig length, max	307451	115205	80308	68117	346047	61418	237840	244377	311058	374789	433364	409751	414955	253362	179407	316779
Contig lenght, min	200	200	200	200	200	200	200	200	200	200	200	200	200	200	200	200
Contig lenght, mean	635	514	512	584	564	518	701	706	879	930	917	960	928	909	625	646
Contig lenght, median	524	441	446	488	423	388	565	571	451	490	492	495	481	476	419	433
Contig lenght, STD	633	448	347	370	1840	741	591	557	2416	2400	2428	2664	2396	2176	1127	1672
Number of bp in contigs	1,28E+09	1,03E+09	7,88E+08	1,28E+09	2,6E+08	2,3E+08	1,59E+09	1,58E+09	7,67E+08	7E+08	7,99E+08	7,12E+08	6,99E+08	7,5E+08	3,59E+08	2,85E+08
Number of contigs	2018347	2002954	1537694	2190673	461470	442807	2264603	2230600	871835	752123	871432	741830	753406	824729	573434	440803

[a] *L50* is defined as count of smallest number of contigs whose length sum makes up 50% of total metagenomic contigs length

[b] *N50* is defined as the sequence length of the shortest contig at 50% of the total metagenomic contigs length

[c] *L90* is defined as count of smallest number of contigs whose length sum makes up 90% of total metagenomic contigs length

[d] *N90* is defined as the sequence length of the shortest contig at 90% of the total metagenomic contigs length
